# Storing Drinking-water in Copper pots Kills Contaminating Diarrhoeagenic Bacteria

**DOI:** 10.3329/jhpn.v30i1.11271

**Published:** 2012-03

**Authors:** V.B. Preethi Sudha, Sheeba Ganesan, G.P. Pazhani, T. Ramamurthy, G.B. Nair, Padma Venkatasubramanian

**Affiliations:** ^1^Centre for Pharmacognosy, Pharmaceutics and Pharmacology, Institute of Ayurveda and Integrative Medicine (formerly Foundation for Revitalisation of Local Health Traditions), 74/2 Jarakabande Kaval, Yelahanka via Attur, Bangalore 560 0106, Karnataka, India; ^2^National Institute of Cholera and Enteric Diseases, P-33, CIT Road, Scheme XM, Beliaghata, Kolkata 700 010, West Bengal, India

**Keywords:** Bacteria, Copper, Diarrhoea, Drinking-water, *Vibrio cholerae*, India

## Abstract

Microbially-unsafe water is still a major concern in most developing countries. Although many water-purification methods exist, these are expensive and beyond the reach of many people, especially in rural areas. Ayurveda recommends the use of copper for storing drinking-water. Therefore, the objective of this study was to evaluate the effect of copper pot on microbially-contaminated drinking-water. The antibacterial effect of copper pot against important diarrhoeagenic bacteria, including *Vibrio cholerae* O1, *Shigella flexneri* 2a, enterotoxigenic *Escherichia coli*, enteropathogenic *E. coli*, *Salmonella enterica* Typhi, and *Salmonella* Paratyphi is reported. When drinking-water (pH 7.83±0.4; source: ground) was contaminated with 500 CFU/mL of the above bacteria and stored in copper pots for 16 hours at room temperature, no bacteria could be recovered on the culture medium. Recovery failed even after resuscitation in enrichment broth, followed by plating on selective media, indicating loss of culturability. This is the first report on the effect of copper on *S. flexneri* 2a, enteropathogenic *E. coli*, and *Salmonella* Paratyphi. After 16 hours, there was a slight increase in the pH of water from 7.83 to 7.93 in the copper pots while the other physicochemical parameters remained unchanged. Copper content (177±16 ppb) in water stored in copper pots was well within the permissible limits of the World Health Organization. Copper holds promise as a point-of-use solution for microbial purification of drinking-water, especially in developing countries.

## INTRODUCTION

Providing safe drinking-water to the majority of the world's population, especially to those in developing countries, is still a major problem. Approximately a billion people lack access to safe drinking-water (1). Water and food contaminated with bacteria, viruses, and protozoa cause infectious diarrhoea. Diarrhoea is one of the leading causes of mortality and morbidity, especially in children of developing countries (2) and claims two million lives each year (3). The major aetiological agents that account for over a million diarrhoeal deaths per year, particularly in developing countries, are enterotoxigenic *Escherichia coli* (ETEC), rotavirus, *Vibrio cholerae*, and species of *Shigella*, which are spread through contaminated water and food or from person to person (4). In India, many states still have outbreaks of cholera. During 1996-2007, at least 222,038 individuals were affected by cholera (5). Shigellosis, also known as acute bacillary dysentery, is associated with complications, such as haemolytic-uraemic syndrome which can be fatal (6). *Shigella flexneri* causes approximately 10% of all diarrhoeal episodes among children aged less than five years (7). Infection with ETEC is associated with traveller's diarrhoea, and the rate of infection is higher in India compared to other developing countries (8). Among the viruses, rotaviruses are the most common cause of diarrhoea in infants and children. In Asia, rotaviruses are responsible for 45% of hospitalizations for severe infantile diarrhoea (9). Microbial quality, though only one of the parameters of safe drinking-water, is a major problem and is a cause of epidemics in developing countries. The existing community interventions to provide safe drinking-water to the people have many shortcomings, and studies have shown that point-of-use (PoU) household interventions contribute to 30-40% reduction in diarrhoeal diseases (10). Moreover, in countries such as India where only 28% of households have piped water (5), PoU interventions are a sustainable way to providing safe drinking-water.

Storing water in copper and silver pots finds mention in ancient texts of Ayurveda for purification of water (11). Our previous study provided laboratory evidence of the antibacterial activity of copper pot in distilled water (12). We had also reported the benefit of using a copper-based device, contrived by us, which was as effective as the pot but at a fraction of the cost (12). Since distilled water is slightly acidic (pH 6.7±0.05) which might enhance copper leaching, we have demonstrated the effect of copper pot in regular drinking-water (pH 7.83±0.4) against important bacterial pathogenic strains that cause diarrhoea.

## MATERIALS AND METHODS

### Bacterial strains

*V. cholerae* O1 IDH 02474 (VC), *S*. *flexneri* type 2a IDH 02196 (SF), *Salmonella enterica* Typhi 500865 (SET), and enterotoxigenic *E. coli* (LT+ST) IDH 01254 (ETEC) were obtained from the National Institute of Cholera and Enteric Diseases (NICED), Kolkata, India. *S*. Paratyphi A B/05 (SPT) was procured from the St. Johns Medical College, Bangalore, India and confirmed at NICED. Enteropathogenic *E. coli* E 2347 (EPEC) was obtained from the Christian Medical College (CMC), Vellore, India.

### Preparation of bacterial cultures

Cultures from the nutrient agar culture-stab were streaked onto selective media, including eosin methylene blue (EMB) agar medium (HIMEDIA, Mumbai, India) for *E. coli* species, xylose lysine dextrose (XLD) medium (HIMEDIA) for *Salmonella* species, Henktoen enteric agar (HEA) medium (Difco, USA) for *Shigella,* and thiosulphate-citrate bile-salts sucrose (TCBS) medium (HIMEDIA) for *V. cholerae,* and were incubated at 37 °C for 16-18 hours in a bacteriological incubator (IN 18 DF, Servewell Instruments Private Limited, Bangalore, India). After incubation, a single colony was picked and inoculated into 2 mL of Luria Bertani broth (Difco) and incubated for 16-18 hours in a bacteriological incubator at 37 °C. This overnight culture was serially diluted in normal saline (NaCl, 0.85%) for inoculation in water.

### Antibacterial activity of copper pot on drinking-water inoculated with enteric pathogens

The experiment procedure followed was essentially as per Sudha *et al*. (12). Copper pots of 2-L capacity (test) purchased from local vendors were thoroughly cleaned and autoclaved each time before use. Presterilized 1-L glass bottles (Schott Duran, Mainz, Germany) acted as controls.

Water was collected from the tap (groundwater, pumped to the overhead tank) from the Microbiology Unit of FRLHT (Foundation for Revitalisation of Local Health Traditions), Bangalore and was autoclaved. The sterilized water was inoculated to ~500 colony-farming unit (CFU)/mL with serially-diluted overnight culture of the diarrhoeagenic bacteria. The same was enumerated by spread plate method on nutrient agar (HIMEDIA). Two litre of inoculated water was poured into copper pots (2×2L) and one litre into each of the two presterilized Schott Duran bottles. After incubation at room temperature (26±2 °C) for 16 hours, 100 μL of samples was withdrawn, after mixing, from each container and plated on nutrient agar for the enumeration of bacteria. Resuscitation of sublethally-damaged cells was monitored by enrichment method (13). Three mL of test or control water sample was mixed with an equal volume of double-strength peptone water (enrichment medium) and incubated for 24 hours at 37 °C. After incubation, the medium was observed for turbidity, and also a loopful of the enriched culture was streaked onto respective selective media as mentioned earlier and observed for growth after incubation for 24 hours at 37 °C. All experiments were conducted three times with duplicates maintained each time.

### Analysis of physical and chemical parameters of water

Tests and controls of the inoculated water were assessed before and after incubation for physicochemical properties, including pH, turbidity, total dissolved solids (TDS), alkalinity, hardness, contents of chlorides and sulphates as per protocols of the Bureau of Indian Standards (14). The pH was measured using a pH meter (DI 707; Digisun Electronics, Hyderabad, India). Copper content was estimated using Spectroquant (Merck, Darmstadt, Germany), a commercially-available, ready-to-use kit, as described in Sudha *et al*. (12).

## RESULTS

### Antibacterial activity of copper pot on drinking-water inoculated with enteric pathogens

VC, SF, ETEC, EPEC, SET, and SPT inoculated into water could not be recovered on the specific growth medium as mentioned in methods (Table 1). In the control glass bottles, on the other hand, the number of bacteria inoculated either remained the same or slightly increased (Table 1). After incubation in the enrichment broth, there was no visible turbidity in the test samples, and no bacteria could be recovered when the enriched cultures were streaked onto selective media. With controls, turbidity in enrichment medium and subsequent growth of bacteria on selective medium were observed (Table 1), where VC exhibited as typical yellow colony on TCBS medium, SF as typical small green colonies on HEA medium, and *Salmonella* species as pink colonies with/without black centre on XLD medium whereas ETEC and EPEC exhibited typical metallic sheen colonies on EMB agar medium. This indicates that the bacteria in the test samples were either completely killed or had lost their culturability on media.

**Table 1.** Effect of overnight storage of tap-water inoculated with diarrhoeagenic bacteria in copper pots and glass bottles

Bacteria inoculated	Before incubation	Copper pots		Glass bottles	
		After incubation		After incubation	
	Dose (CFU/mL)	Bacterial count (CFU/mL)	Enrichment culture	Bacterial count (CFU/mL)	Enrichment culture
*V. cholerae* O1 IDH 2474	506±11	No growth	Not detected	516±11	Detected
*S. flexneri* 2a IDH 02196	533±28	No growth	Not detected	530±26	Detected
ETEC IDH 01254	513±23	No growth	Not detected	866±83	Detected
EPEC E2347	506±11	No growth	Not detected	600±10	Detected
*S. enterica* Typhi 500865	170±53	No growth	Not detected	109±66	Detected
*S.* Paratyphi A	453±109	No growth	Not detected	361±67	Detected

CFU=Colony-forming unit;

EPEC=Enteropathogenic *Escherichia coli*;

ETEC=Enterotoxigenic *Escherichia coli*

### Physical and chemical parameters

The level of copper that had leached into the test samples was 177±16 ppb which was well within the WHO limit of 2000 ppb (Table 2). TDS, alkalinity, hardness, contents of chlorides and sulphates remained the same before and after incubation in both test and control samples, except that the pH had slightly increased from 7.83±0.4 to 7.93±0.3 in the test samples (copper pot) after incubation for 16 hours (Table 2).

## DISCUSSION

None of the test pathogens was recovered from drinking-water stored in copper pots even after enrichment culture. This is the first report on the antibacterial activity of copper against pathogenic strains of SF, EPEC, and SPT. Copper pot is as active in regular drinking-water (pH 7.83±0.4) as that reported by us earlier (12) in distilled water (pH 6.7±0.05), and the level of copper leached in the former is far less (177±16 ppb) than that in distilled water (~420 ppb). Other studies have shown that copper vessel is lethal to *E. coli* in water at different pH and temperature conditions, with the fastest inactivation occurring as the pH shifts away from neutrality and 35 °C (15). Copper has also been shown to act, to a greater or lesser extent, on *E. coli* in the presence of organic and inorganic constituents in water (16). In laboratory experiments, copper has been shown to kill meticillin-resistant *Staphylococcus aureus* (17), *Campylobacter jejuni,* and *S*. *enterica* (18). Findings of these studies suggest that copper can act on a range of organisms under different conditions. It is still important to test the effect of copper on various sources of drinking-water under different field conditions. Safety of leached copper does not appear to be an issue since studies have shown that the current WHO guideline of 2 mg Cu/L is safe (19,20), and the levels leached in the study were 1/20^th^ of the permissible limits. It has been observed in the present study that the other physicochemical parameters of drinking-water remain unchanged after copper intervention, which makes them amenable for public use.

We observed that the unrecovered bacteria in the test samples did not get resuscitated even after enrichment and plating on selective media. This indicates that they have lost culturability on non-selective medium and on enrichment and selective media. However, we still need to confirm whether they have transformed into the viable but non-culturable (VBNC) state. VBNC is a state in bacteria where the cells do not grow onto routinely-employed media but are still viable (21). VBNC bacteria have been studied using several methods, including alteration in temperature (22), use of enrichment medium (23), changes in the growth medium, using chick embryo yolk sack, passage through rabbit illeal loop (13), or co-culturing with eukaryotic cell cultures (21). VBNC bacteria have been observed using viable stains (22) and microscopy (21,24,25).

**Table 2.** Physicochemical quality of tap-water before and after incubation in copper pot and in glass bottles

Parameter	Permissible limit (BIS/WHO*)	Before incubation	After incubation	
			Test	Control
Alkalinity (mg/L)	600	25	25	25
Hardness (mg/L)	600	280	280	280
Turbidity (NTU)	10	0.47	0.47	0.47
TDS (mg/L)	2,000	700±49.5	655±35.4	690±14
Chlorides (mg/L)	1,000	35.45	35.45	35.45
Sulphates (mg/L)	400	86.5	86.5	86.5
pH	8.5-9.0	7.83±0.4	7.93±0.3	7.83±0.4
Copper content (mg/L)*	2	<DL 0.1	0.177±0.016	<DL

*Detectable limit=0.02 mg/L;

BIS=Bureau of Indian Standards;

DL=Detecting limit;

NTU=Nephelometric turbidity unit;

TDS=Total dissolved solids;

WHO=World Health Organization

Studies have shown that copper surfaces completely kill bacteria. *E. coli* inoculated on to copper coupons were completely killed. The studies concluded that the copper ions brought about complete killing of bacteria by membrane damage (26). However, the mechanism of action of copper on bacteria is not completely understood.

Although studies have shown the merits of copper surfaces for their use in improving public hygiene in healthcare facilities, the potential use of copper for the purification of drinking-water, especially in developing countries, has not been widely studied. Therefore, results of our study indicate that copper holds potential to provide microbially-safe drinking-water to the rural masses in developing countries. The use of copper pots in Indian households is common and is, therefore, likely to be socially accepted by the people. Its functioning is not dependent on fuel, electricity, replaceable filters, intensity of sunlight, etc. to operate or maintain it; it is simply a passive storage of water. This takes into account the conditions prevailing in rural villages and the urban slums of developing countries. The health benefit that can be achieved by using copper pot as a PoU water-purification device will far outweigh the cost of the pot, if divided over the members in a rural family, especially as it will be a one-time investment with no recurring costs. However, it is important to challenge its use under real-life conditions in the dynamics of the target households in developing countries to fully understand the limitations.

## ACKNOWLEDGEMENTS

The study was supported by the ETC CAPTURED Programme, The Netherlands (Grant No. DGIS/D). The authors thank Mr. Darshan Shankar, Advisor, IAIM, for his constant encouragement.
